# Hand hygiene opportunities in neonatal intensive care: a multicenter observational study to calibrate group electronic monitoring systems

**DOI:** 10.1017/ice.2025.36

**Published:** 2025-05

**Authors:** Eugene Lee, Stacey Clark, Paige Reason, Sarah Khan, Sabrina Fan, Michelle Li, Alex Cen, Asaph Rolnitsky, Alexander Kiss, Dominik Mertz, Jerome A. Leis

**Affiliations:** 1 Sunnybrook Health Sciences Centre, Toronto, ON, Canada; 2 Hamilton Health Sciences, Hamilton, ON, Canada; 3 Faculty of Health Sciences, McMaster University, Hamilton, ON, Canada; 4 Centre for Quality Improvement and Patient Safety, University of Toronto, Toronto, ON, Canada; 5 Temerty Faculty of Medicine, University of Toronto, Toronto, ON, Canada

## Abstract

Observers were randomized to time and location across two different Neonatal Intensive Care Units (NICUs) to count hand hygiene opportunities (HHOs). Mean hourly HHO was lower at night and during use of precautions, and higher in shared rooms. HHO benchmarks can support implementation of group electronic monitoring systems in NICUs.

## Introduction

Despite remaining the gold standard for hand hygiene measurement, direct observation remains an inaccurate way of benchmarking hand hygiene performance.^1^

Group electronic monitoring systems are automated solutions with potential to overcome these limitations.^2^ These systems incorporate sensors on all alcohol and hand-based dispensers to measure hand hygiene events but require an accurate assessment of daily hand hygiene opportunities (HHO) to calculate an estimated compliance. This denominator has been determined across multiple different adult populations but data is lacking in pediatrics.^2–5^

Prematurely born infants admitted to neonatal intensive care units (NICU) are highly vulnerable to healthcare-associated infection.^6^ We conducted this multicenter study to assess HHO rates and inform future implementation of group electronic monitoring systems in this population.

## Methods

### Study setting and patient population

The study was conducted across two NICUs in Ontario, Canada: (1) Sunnybrook Health Sciences Centre with 42 level 3 beds, and (2) McMaster Children’s Hospital with 56 level 3 beds and 16 level 2 beds. Research ethics review was not required because the project met criteria for exemption based on institutional process that confirmed it was deemed quality improvement and not human subject research.

### Observer training and randomization to location and time

University students were trained to identify HHO using the Public Health Ontario (PHO) methodology as done previously.^4^ This methodology applied to NICU includes Moment 1A before touching patient environment (ie incubator), Moment 1B before patient contact, Moment 2 prior to an aseptic procedure, Moment 3 after bodily fluid exposure risk, and Moment 4 after contact with patient and/or patient environment. Excluding moment 1A corresponds to the World Health Organization (WHO) Five Moments with Moments 4 and 5 collapsed as a single Moment 4.

Observers were randomized to bed number and 4-hour observation blocks to ensure equal representation of location and time periods. If an observer could not attend a scheduled session, that session was moved to the end of the observation schedule to mitigate any gaps in the data.

### Data collection

Observations were completed from 1 August, 2022 to 30 April, 2023 to achieve target sample size of 100 hours per site. Data abstraction form included entry/exit times, HHOs observed, day of week (weekday/weekend), hour of day, healthcare worker type (nurse, physician, allied health worker, other), presence of transmission-based precautions (yes/no), and room type (open pod, private room, shared room). Allied health workers included environmental services, health care aid, lactation specialist and diet technician, respiratory therapist, patient transporter, and physiotherapist. If curtains were drawn, observers asked the healthcare worker about tasks performed and recorded any HHOs that would have occurred. If the inpatient was temporarily off the unit, observers remained assigned to this bedside to avoid over-estimating HHOs. In case of discharge occurring during the observation period, the observer ended observation of this bed and moved to the nearest bedside to continue observations. When blended moments occurred, defined as consecutive HHOs without patient or environment contact in between, only the first HHO was recorded.

### Statistical analysis

Counts and percentages for categorical variables and means with standard deviations for continuous variables, were calculated. The primary outcome was the mean HHO per patient hour, calculated as a weighted mean across 24 hours, with each hour contributing equally toward the mean. Due to non-parametric distribution, mean HHO was compared between sites using Mann-Whitney test. Combined site HHO was broken down by healthcare worker type, day/night (07:00 A.M. – 07:00 P.M. vs 07:00 P.M. – 07:00 A.M.). A Poisson regression model was used to assess predictors of HHO while adjusting for time nested within each site, day of week (weekday vs weekend), time of day (day vs night), unit design (open pod vs private rooms vs shared rooms), and presence of transmission-based precautions. Multicollinearity was excluded (tolerance >0.4). The model adjusted for overdispersion and presented results as incidence rate ratios (IRR) and their associated 95% confidence intervals. Analyses were carried out using SAS Version 9.4 (SAS Institute, Cary, NC, USA).

## Results

There were 277 patient hours of observation including 136 (49%) at site A and 141 (51%) at site B. Among 2140 HHOs, there were 639 (30%) moment 1a, 339 (16%) moment 1b, 153 (7%) moment 2, 196 (9%) moment 3, and 813 (38%) moment 4. The breakdown of HHO by health care worker was 1954 (91%) nurses, 38 (2%) physicians, 124 (6%) allied health workers, and 24 (1%) other.

**Figure 1.**
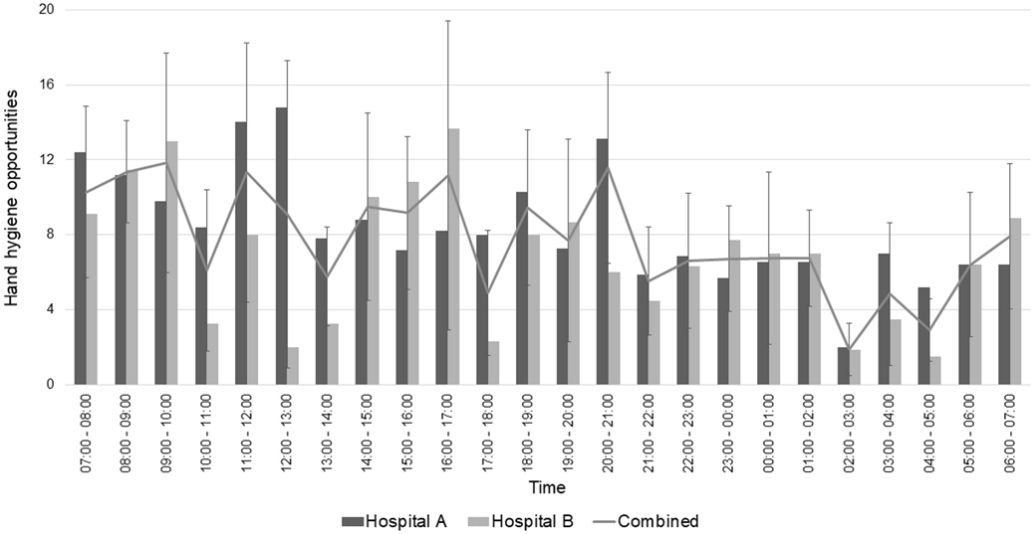
Mean hourly hand hygiene opportunities per bed hour over 24-hour period across two Canadian Neonatal Intensive Care units.

The hourly breakdown by hospital is depicted in Figure 1. Mean HHO per patient hour was no different between hospitals A and B (8.33, 95% CI, 7.05–9.61 vs 6.84, 95% CI, 5.35–8.34; *P* =.2). Table 1 summarizes the combined mean HHO per patient hour, during the day, at night, weekdays and weekends, by either PHO or WHO methodology.

**Table 1.** Mean hand hygiene opportunities (HHO) in two Canadian Neonatal Intensive Care Units based on Public Health Ontario and World Health Organization classified moments of hand hygiene

HHO per patient hour	Public health Ontario	World Health Organization
Overall	7.74 (95% CI, 6.57–8.91)	5.42 (95% CI, 4.51–6.33)
Daytime^a^	9.17 (95% CI, 7.67–10.67)	6.40 (95% CI, 5.14–7.67)
Nighttime^b^	6.31 (95% CI, 4.75–7.87)	4.44 (95% CI, 3.24–5.63)
Weekday	7.51 (95% CI, 6.04–8.98)	5.25 (95% CI, 4.15–6.34)
Weekend	7.76 (95% CI, 6.27–9.25)	5.40 (95% CI, 4.21–6.59)

a Daytime, 07:00 A.M. – 07:00 P.M.

b Nighttime,07:00 P.M. – 07:00 A.M.

Daytime was associated with significantly higher HHOs compared to night (IRR 1.46, 95% CI, 1.13–1.89; *P* =.004) while there was no significant difference on weekends (IRR 0.93, 95% CI, 0.85–1.03; *P* =.17). Both private rooms (IRR 0.76, 95% CI, 0.64–0.90; *P* =.001) and open pod (IRR 0.73, 95% CI, 0.58–0.90; *P* =.005) were associated with fewer HHOs compared to shared rooms. Use of transmission-based precautions was associated with fewer HHOs (IRR 0.78, 95% CI, 067–0.91; *P* =.002).

## Discussion

In this multicenter study, we found a similar hourly HHO across two different NICUs. These rates may serve as a generalizable benchmark for calibrating group electronic monitoring systems in this unique setting.

Measurement of hand hygiene in NICU continues to rely on direct observation across most institutions. Some NICUs have implemented badge-based systems that pick up proximity to hand sanitizers and these do not require an accurate denominator of HHO.^7,8^ The downside of these systems is that they can miss data when badges are not worn, and discordant measurements may occur when in proximity without an HHO. Group electronic monitoring systems capture all hand hygiene events without measuring individual-level compliance and have been used broadly in adult populations.^2,5^

To expand the use of group monitoring systems in the NICU setting, our findings suggest that HHO benchmarks from adult studies cannot be applied to this population. We observed higher HHOs in NICU compared to adult inpatient units (3–3.5 per bed hour) yet lower compared to adult intensive care units (7.4–8.6).^3–5^ The average HHO in our study also falls within the 95% confidence intervals of one prior observational study that included 31 hours of observation in the NICU.^9^

Weekends were not associated with decreased HHOs in our study, similar to adult populations in Canada, the United States, and Australia.^3,4,10^ Shared rooms were associated with higher HHOs possibly due to increased interaction within these spaces. Conversely, patients in precautions had fewer HHOs possibly due to reduced patient interactions, or batching of tasks by staff to minimize entry into isolation rooms.

This study has several important limitations. First, it is an observational study where HHO could have been confounded by additional unmeasured factors. Patient acuity has been shown to correlate with HHO in prior studies.^3^ Second, although we randomized observers to day of the week, fewer weekend observations occurred due to scheduling challenges. Despite this, the combined sample size of weekend observations remains similar to previous studies.^3,4^ Third, the NICUs observed in Canadian context may not be applicable to other countries, and further validation is needed.

The number of HHO in NICU is unique to this setting. Incorporation of this benchmark into group electronic monitoring solutions may support wider adoption for measuring hand hygiene adherence in NICUs.
